# Continuous Preparation of Hollow Polymeric Nanocapsules Using Self-Assembly and a Photo-Crosslinking Process of an Amphiphilic Block Copolymer

**DOI:** 10.3390/molecules22111892

**Published:** 2017-11-03

**Authors:** Xuan Don Nguyen, Hyeong Jin Jeon, Van Tien Nguyen, Dong Hyeok Park, Taeheon Lee, Hyun-jong Paik, June Huh, Jeung Sang Go

**Affiliations:** 1 School of Mechanical Engineering, Pusan National University, 2, Busandaehak-ro 63 beon-gil, Geumjeong-gu, Busan 46241, Korea; nguyenxuandon@pusan.ac.kr (X.D.N.); hjjeon@pusan.ac.kr (H.J.J.); nguyenvantien@pusan.ac.kr (V.T.N.); dhpark90@pusan.ac.kr (D.H.P.); 2 Department of Polymer Science & Engineering, Pusan National University, 2, Busandaehak-ro 63 beon-gil, Geumjeong-gu, Busan 46241, Korea; taeheonlee1379@gmail.com; 3 Department of Chemical & Biological Engineering, College of Engineering, Korea University, Anam-Dong, Seongbuk-Gu, Seoul 02841, Korea; junehuh@korea.ac.kr

**Keywords:** hollow nanocapsules, block copolymer, OLED, light trapping

## Abstract

This paper presents a fabrication method of hollow polymeric nanocapsules (HPNCs). The HPNCs were examined to reduce light trapping in an organic light emitting diodes (OLED) device by increasing the refractive index contrast. They were continuously fabricated by the sequential process of self-assembly and photo-crosslinking of an amphiphilic block copolymer of SBR-*b*-PEGMA, poly(styrene-r-butadiene)-*b*-poly(poly(ethylene glycol) methyl ether methacrylate) in a flow-focusing microfluidic device. After the photo-crosslinking process, the produced HPNCs have a higher resistance to water and organic solvents, which is applicable to the fabrication process of optical devices. The morphology and hollow structure of the produced nanocapsules were determined by transmission electron microscopy (TEM), scanning electron microscopy (SEM), and atomic force microscopy (AFM). Also, their size control was examined by varying the ratio of inlet flow rates and the morphological difference was studied by changing the polymer concentration. The size was measured by dynamic light scattering (DLS). The refractive index of the layer with and without the HPNCs was measured, and a lower refractive index was obtained in the HPNCs-dispersed layer. In future work, the light extraction efficiency of the HPNCs-dispersed OLED will be examined.

## 1. Introduction

**Figure 1:**
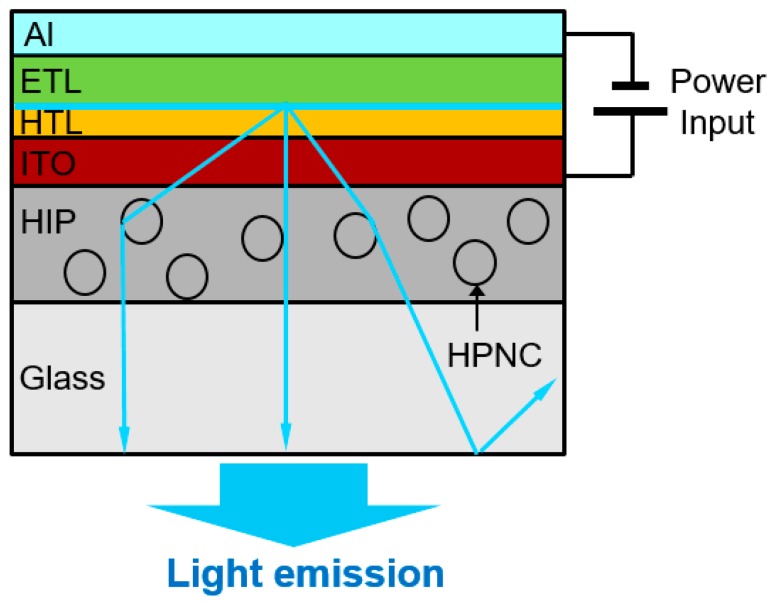
Cross-sectional view of organic light emitting diodes (OLED) incorporated with hollow polymeric nanocapsules (HPNCs).

Organic light emitting diodes (OLED) have drawn attention for display applications as a thin film light source. Recently, the problem of light trapping in OLED devices has been addressed. Thirty percent of the light is reflected in the interface of glass and air and only 20% of the generated light can escape as useful light. Since the largest portion of light is trapped within the organic-ITO (Indium Tin Oxide) layer, as shown in Figure 1, the most efficient method to improve outcoupling efficiency (hereafter, OCE) has been introduced to insert optical scattering structures in close proximity to the ITO layer [1,2,3,4].

Various optical scattering structures have been developed. A high index grating of silicon nitride was used to extract the trapped light and fabricated by using nano-imprinting technology [5,6]. Also, the nanostructures were randomly patterned on the ITO by using wet chemical etching [7]. In addition, buckling structures with random periods were reported [8]. However, the periodic structures of the grating caused spectral shift, so that the viewing angle was constrained.

To this end, optical scattering using nanoparticles dispersed in the polymer layer was recently examined [9]. The scattering layer was fabricated simply by spin-coating, and 1.5 times enhancement of the OCE was achieved in comparison with ordinary OLED. The optical scattering efficiency of the nanoparticle-dispersed polymer layer mainly is obtained by the refractive index contrast between the nanoparticles and the polymer. This means that an additional increase in the index contrast can enhance the OCE and, as a result, the light trapping can be reduced.

HPNCs consisting of polymer shells and air can provide the largest index contrast achievable in nanoparticle-dispersed polymers. HPNCs have demonstrated a high potential in various uses, such as biomedical applications [10], controlled drug release [11], and medical therapeutics [12]. To prepare them, a mini-emulsion polymerization process has been reported [13,14], using styrene and methyl methacrylate as the monomers. Also, two efficient and robust techniques called polymerization-induced self-assembly (PISA) [15,16] and temperature-induced morphological transformation (TIMT) can be used [17,18]. Although these facile and versatile techniques have made significant contributions for industrial and biomedical applications, the synthetic procedures are complicated and require many steps.

In this work, a continuous online preparation of HPNCs with uniform size distribution was achieved by combining the sequential processes of self-assembly and photo-crosslinking of an amphiphilic block copolymer in a flow-focusing microfluidic device. The amphiphilic block copolymers have shown unique properties and numerous potential applications due to their ability to self-assemble in solution to form various morphologies including spherical micelles, cylindrical micelles, and vesicles [19,20,21,22]. Specifically, the self-assembly of the amphiphilic block copolymers in the microfluidic focused flow has been investigated to produce spherical polymeric micelles [23,24]. Fast mixing by diffusion through the interface of the laminated flow in the microfluidic channel was utilized. The polymeric micelles were formed due to the solvent exchange process, when the polymer-dissolved organic solution was mixed with a non-solvent fluid. The microfluidic synthesis exhibited uniform size distribution and preparation stability. However, most of the reported HPNCs have low resistance to organic solvents and water because of their biodegradable property. As a result, their application to OLED devices is limited.

To apply HPNCs for the fabrication process of an OLED device, high resistance to water and organic solvents should be guaranteed. In this work, SBR-*b*-PEGMA block copolymers were synthesized. To form the HPNCs, a consecutive process involving the self-assembly of the SBR-*b*-PEGMA block copolymers and photo-crosslinking was performed. For photo-crosslinking, the materials require photo-crosslinkable materials, photoinitiators, and crosslinking agents. A high-speed photo-crosslinking process was employed for the SBR-*b*-PEGMA [25].

## 2. Results and Discussions

### 2.1. Continuous Preparation of HPNCs

**Figure 2:**
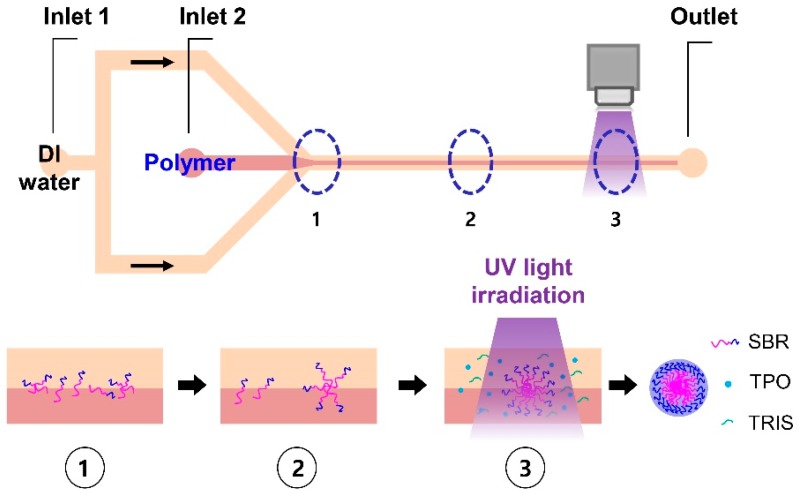
Schematic diagram of experiment to fabricate HPNCs: ① Dispersion and nucleation; ② Growth through aggregation; ③ Self-assembly and photo-crosslinking.

The process of fabricating the HPNCs was conducted in a flow-focused microfluidic channel with two inlets and one outlet, as illustrated in Figure 2. A mixture of 9.6 mg of SBR-*b*-PEGMA (*M_n_* = 33,000 g/mol), 10 μL of the crosslinking agent TRIS (Trimethylolpropane mercaptopropionate), 5 mg of the photoinitiator Lucirin TPO (Diphenyl(2,4,6-trimethylbenzoyl)phosphine oxide), and 50 μL of Tween 80 were dissolved in 3 mL of Tetrahydrofuran (THF), and the mixture was introduced into the central inlet of the microchannel at a flow rate of 40 µL/min. The dissolved polymer stream was focused by DI (De-ionized) water injected from the side inlets at a flow rate of 80 µL/min. At the interface of the laminated flows which subsequently formed in the microchannel, polymeric micelles were self-assembled due to the solvent exchange process.

UV source (ANUP5204, Panasonic, Osaka Prefecture, Japan) was positioned 5 mm away from the outlet and irradiated over the 10-mm-long microchannel to initiate the photo-crosslinking process between the crosslinking agent and photoinitiator, which occurred under the UV irradiation. After the photo-crosslinking process, the HPNCs were stable, insoluble, and strongly resistant to water and organic solvents. They were collected at the outlet for further investigation.

### 2.2. The Mixing Time for Hydrodynamic Focusing Flow

**Figure 3:**
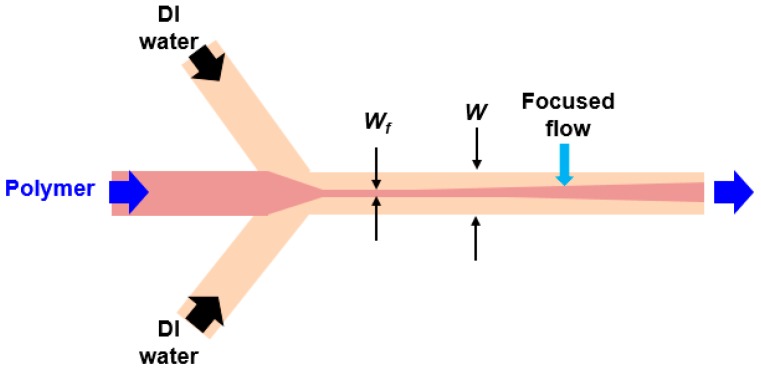
Schematic of hydrodynamic focusing flow.

When the dissolved polymer stream flows through the junction of the microchannels and meets the water stream, a stable hydrodynamic focusing flow is generated along the length of the microchannel. A schematic of this hydrodynamic focusing flow is shown in Figure 3. By adjusting the width of the focused flow (*w_f_*), the mixing process in the microchannel can be well-controlled, based on diffusion. The diffusion of the solvent and non-solvent molecules through the interface between the laminated flows results in diffusive mixing, and a nanoprecipitation process occurs [23,26]. The polymeric micelles are subsequently formed. Their size characteristics are defined by the ratio of flow rates, the aspect ratio of the microchannel, and the viscosity and fluidic conditions, as reported in Reference [24].

The theoretical mixting time can be described as follows [23,27].

$$\tau_{mix}\sim \frac{w_{f}^{2}}{4D}\approx \frac{w^{2}}{9D}\frac{1}{{\left(1+1/R\right)}^{2}}$$

where *D* is the diffusion coefficient of the solvent, *w_f_* is the width of the focused flow, *w* is the channel width, and *R* is the ratio of the polymeric flow rate to the flow rate of the water. For a flow rate ratio of 0.5, a diffusion coefficient of THF in water of 9.88 × 10^−10^ m^2^/s, and a microchannel width of 50 μm, the mixing time was calculated to be 0.03 s. The longer the mixing time is, the larger the nanocapsule size that can be obtained.

**Figure 4:**
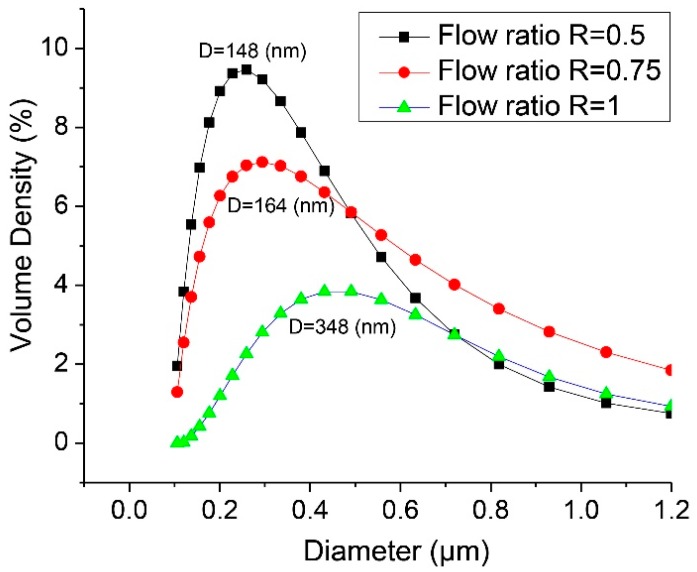
Size distribution of nanocapsules for the inlet flow rates. D, mean diameter.

To examine the effect of the mixing time on the size of HPNCs, the ratio of the inlet flow rates was varied, as shown in Table 1. The smaller ratio of the flow rates results in the shorter mixing time. As shown in Figure 4, the size of the HPNCs was measured by dynamic light scattering (DLS) (Mastersizer 3000E, Malvern Instruments, Malvern, UK). The size of the HPNCs increases with the increasing ratio of the flow rates, which results in a longer mixing time.

### 2.3. Flow Visualization and Size Distribution of HPNCs

**Figure 5:**
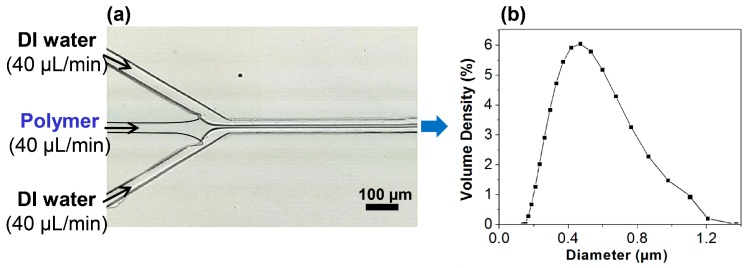
(**a**) Visualization of the focusing flow in the microchannel; (**b**) Size distribution of HPNCs.

A focused lamination flow was formed in the microfluidic device and visualized using a high-speed CCD (Charged Coupled Device) camera connected to a microscope (BX-51, Olympus, Tokyo, Japan). In order to examine the size distribution of the HPNCs, the sample solution was analyzed using dynamic light scattering (DLS) (Mastersizer 3000E, Malvern Instruments, Malvern, UK). The visualization of the flow in the microchannel and the size distribution of the produced HPNCs are shown in Figure 5. A strong focusing flow was obtained and the mean diameter of the HPNCs was measured to be 284.0 nm.

### 2.4. TEM, SEM, and Atomic Force Microscopy (AFM) Characterization of HPNCs

**Figure 6:**
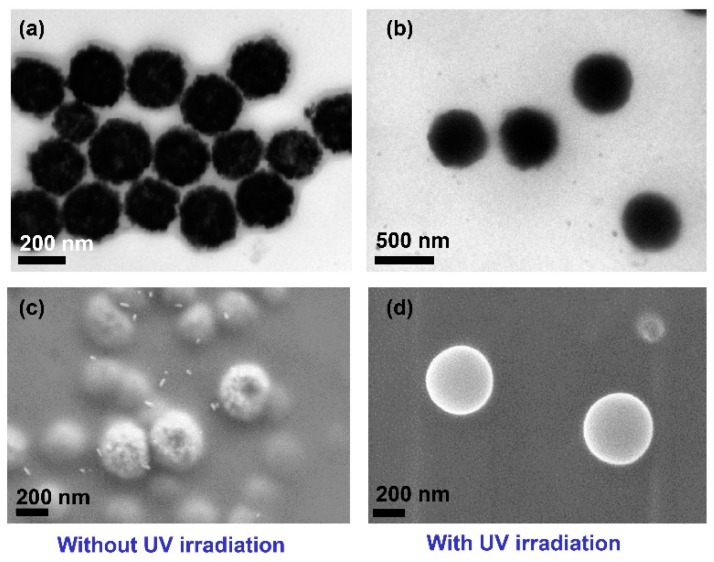
TEM (**a**,**b**) and SEM (**c**,**d**) images of HPNCs prepared with and without the photo-crosslinking process.

**Figure 7:**
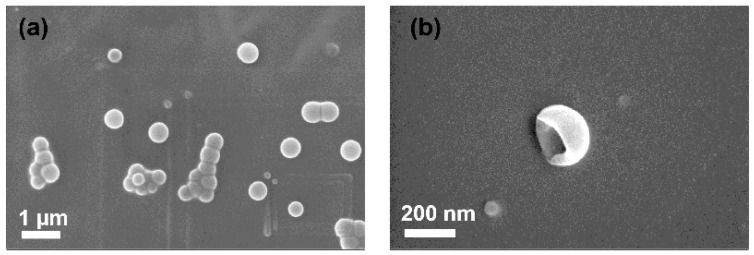
(**a**) SEM image of the produced HPNCs; (**b**) Confirmation of hollow nanostructure.

TEM (H-7600 Transmission Electron Microscope, Hitachi High-Technologies Corporation, Tokyo, Japan) and SEM (Zeiss Supra 25 FE-SEM, Carl Zeiss AG, Oberkochen, Germany) were used to characterize the HPNCs, and the images are shown in Figure 6. The HPNCs prepared by photo-crosslinking show a more spherical and smooth surface compared to those prepared without the photo-crosslinking process. The partly flat HPNCs are also shown in Figure 7, which proves the hollow structure of the nanoparticles.

**Figure 8:**
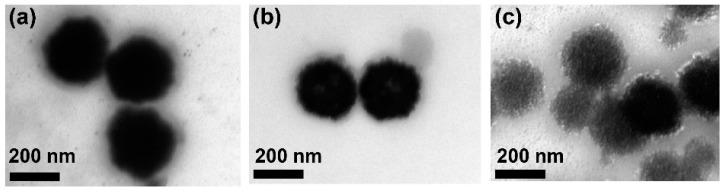
TEM images of HPNCs for various polymer concentrations: (**a**) 5 mg/mL; (**b**) 3 mg/mL; (**c**) 1 mg/mL.

The effect of the polymer concentration on the morphology of the HPNCs was also conducted by taking TEM images. The TEM images in Figure 8 show that the lower the polymer concentration is, the less condensed the morphology of HPNCs is obtained.

**Figure 9:**
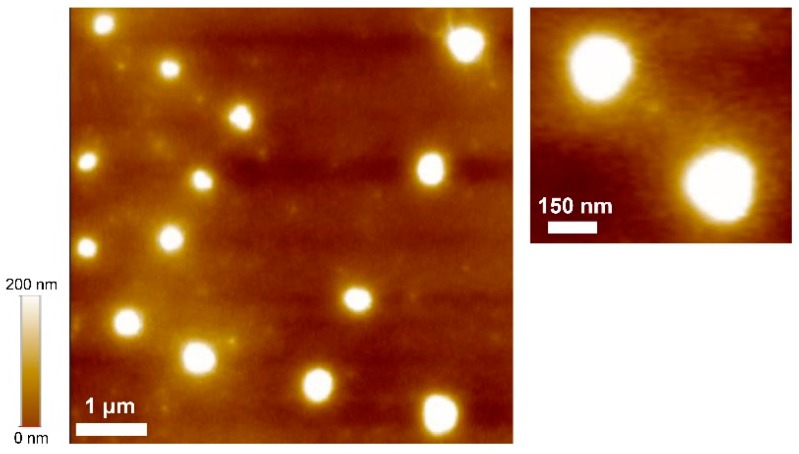
Atomic force microscopy (AFM) images of the produced HPNCs.

AFM (XE-7, Park Systems Co., Suwon, Korea) was also used to characterize the produced HPNCs, and the results are shown in Figure 9. The AFM images also proved the nanosize distribution of the HPNCs corresponding to the DLS measurement and the TEM and SEM images shown above.

### 2.5. Measurement of the Refractive Index of HPNCs

**Figure 10:**
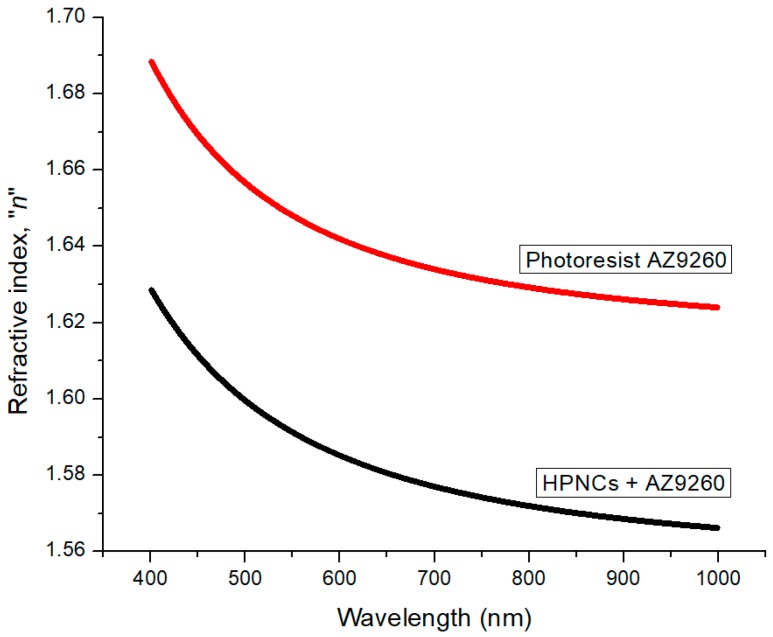
Refractive indices of the photoresist AZ9260 and the HPNCs-dispersed photoresist AZ9260.

To examine the optical property of HPNCs, the refractive index was measured by using a Spectroscopic Ellipsometer (M-2000 Ellipsometer, J.A. Woollam Co., Lincoln, NE, USA). The produced HPNCs were mixed with the photoresist AZ9260 and spin-coated with a thickness of 2.2 ± 0.1 µm on a silicon wafer. Also, the pure photoresist AZ9260 was spin-coated. The refractive index of the spin-coated photoresist AZ9260 mixed with the HPNCs should be smaller than that of the AZ9260 layer. Figure 10 and Table 2 show the comparison of the theoretical and measured refractive indices of the AZ9260 layer with and without the HPNCs. The refractive index of the HPNCs-dispersed AZ9260 layer is measured to be smaller than that of the AZ9260 layer, which results in a higher index contrast for the application of OLED.

### 2.6. Resistance Test of HPNCs to an Organic Solvent

The solvent resistance of the HPNCs was tested in the organic solvent of tetrahydrofuran (THF), which is commonly used as a dissolving solution. It was kept for seven days, during which the weight was measured periodically. The weight of the HPNCs was 2.3 mg before dispersing in THF. After evaporating all of the solvent, the weight of the HPNCs was 2.32 mg. This means that the prepared HPNCs are resistant to the solvent.

## 3. Materials and Methods

### 3.1. Materials

Lucirin TPO, TRIS, and Tween 80 were purchased from Sigma-Aldrich Chemicals (St. Louis, MO, USA) and used as received. THF was purchased from Daejung Chemicals and Metals (Gyeonggi-do, South Korea) and used as received. Styrene (Junsei Chemicals, Tokyo, Japan, 99.5%) was purified by vacuum distillation with CaH_2_. Copper(I) bromide (CuBr; Sigma-Aldrich Chemicals, 98.0%) was purified by stirring with glacial acetic acid, followed by filtering and washing the solid thrice with methanol and twice with diethyl ether. The solid was vacuum-dried for two days. PEGMA (*M_n_* = 300 g/mol; Sigma-Aldrich) was purified by passing it through a column filled with basic alumina to remove the inhibitors. Cyclohexane (Sigma-Aldrich, 99%) was dehydrated by vacuum distillation. Ethylene glycol (99.8%), *n*-butyl lithium (2.0 mol/L in cyclohexane), *N*,*N*,*N*′,*N*″,*N*″-pentamethyldiethylenetriamine (PMDETA; 99%), α-bromoisobutyryl bromide (98%), and copper(II) bromide (CuBr_2_; 98.0%) were purchased from Sigma-Aldrich and used as received. 1,3-Butadiene and ditetrahydrofurylpropane (DTHFP) were obtained from Kumho Petrochemical and used as received. The synthetic rubber used in the experiment was SBR 1739 (Kumho Petrochemical; styrene content of 40 wt %; oil extended in 37.5 phr (parts per hundred rubber by weight)).

### 3.2. Synthesis of SBR-b-PEGMA by Atom Transfer Radical Polymerization (ATRP)

**Figure 11:**
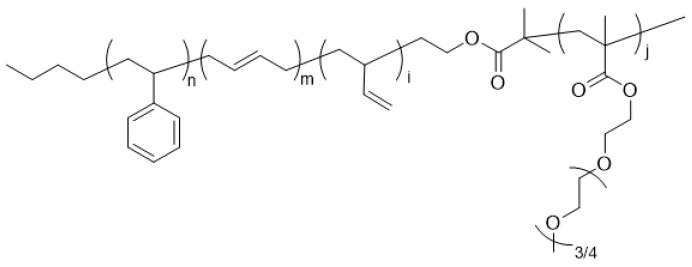
Structure of SBR-*b*-PEGMA.

SBR-*b*-PEGMA, shown in Figure 11, contains a double bond that is free to participate in the photo-crosslinking process, and was synthesized from the macroinitiator pSB-Br. First, the macroinitiator pSB-Br was prepared as follows. In a nitrogen-purged high-pressure reactor, ditetrahydrofurylpropane (DTHFP, 0.30 mL, 1.14 mmol) was dispersed in dried cyclohexane (920 mL). Then, styrene (48 g, 0.46 mol), butadiene (72 g, 1.33 mol), and n-butyl lithium (8.0 mL, 16 mmol) were added using an airtight syringe at 30 °C. The reaction mixture was stirred until a maximum temperature was reached (85 °C). After 5 min of stirring, ethylene oxide (3.0 g, 50 mmol) and α-bromoisobutyryl bromide (5.0 mL, 15.6 mmol) were sequentially added. The result was precipitated in methanol and vacuum-dried at 40 °C (*M_n,GPC_* = 11,500 g/mol, *M_n,NMR_* = 6700 g/mol, PDI (Polydispersity index) = 1.08).

SBR-*b*-PEGMA, which was then required to have amphiphilic properties, was prepared as follows. A cleaned and dried 200-mL Schlenk flask was charged with PEGMA (50 mL, 175 mmol), anisole (100 mL), and PMDETA (52.4 µL, 2.50 × 10^−1^ mol). The mixture in the flask was degassed via three freeze–pump–thaw cycles. During the final cycle, the flask was filled with nitrogen; then, CuBr (40.2 mg, 2.80 × 10^−1^ mol), CuBr_2_ (15.6 mg, 7.0 × 10^−2^ mol), and pSB-Br (2.70 g, 3.50 × 10^−1^ mmol) were quickly added to the frozen mixture. The flask was sealed with a glass stopper, and then evacuated and backfilled with nitrogen three times before immersing it in an oil bath at 40 °C. After 3 h, the polymerization reaction was quenched by exposing the flask to air. The mixture was dissolved in tetrahydrofuran, passed through a column of neutral alumina, and then precipitated into hexanes. After filtering and vacuum-drying the product, a light yellow powder was isolated. The molecular weight of the obtained polymer was determined using gel permeation chromatography (GPC) (*M_n,GPC_* = 33,000 g/mol, *M_n,NMR_* = 31,000 g/mol, PDI = 1.34).

**Figure 12:**
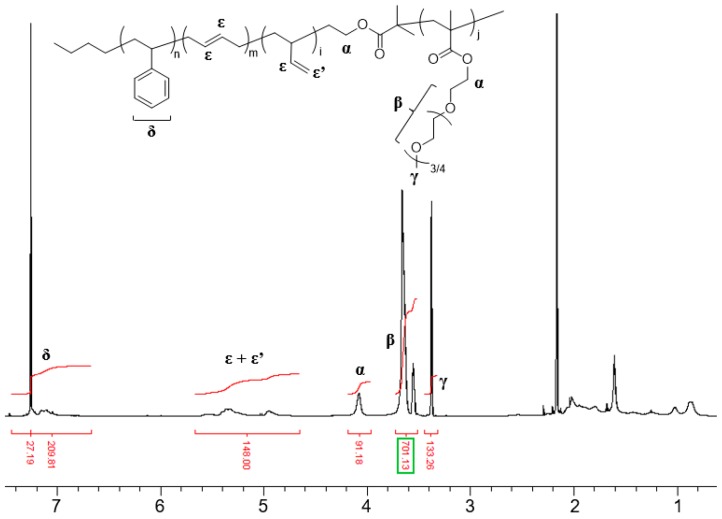
^1^H-NMR spectrum of SBR-*b*-PEGMA.

The synthesized SBR-*b*-PEGMA was characterized by ^1^H-NMR to determine the molecular weight and PEGMA composition in the polymer chain. The ^1^H-NMR spectrum of the synthesized SBR-*b*-PEGMA shown in Figure 12 indicates that the ATRP had proceeded well. The peaks at 7.40–6.58 ppm correspond to the aromatic protons of the styrene units in the polymer. The peaks at 5.0–4.5 ppm and 5.7–5.0 ppm can be assigned to the methenyl protons (–CH=CH_2_) of the 1,2-addition of butadiene and the vinyl protons (–CH=CH–) of the 1,4-addition (overlapping region) and the methenyl (–CH=CH_2_) protons of the 1,2-addition, respectively. The composition of the styrene and butadiene in SBR-*b*-PEGMA can be determined by comparing the peaks at 7.40–6.58 ppm with those at 7.5–4.5 ppm. The peak at 3.3 ppm in the ^1^H-NMR spectrum can be assigned to the methoxy protons (OCH_3_) using the aromatic protons in styrene as a standard.

### 3.3. Fabrication of Flow-Focusing Microfluidic Device

**Figure 13:**
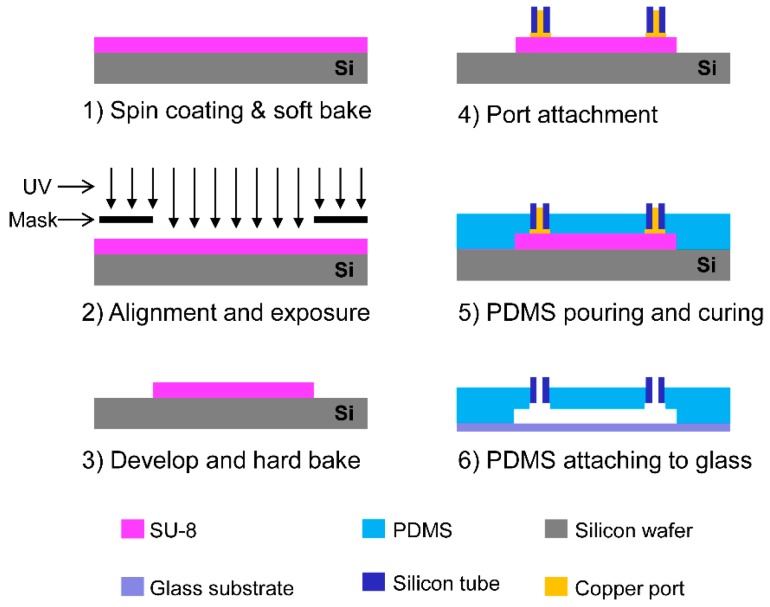
Steps in the fabrication of the microfluidic device.

**Figure 14:**
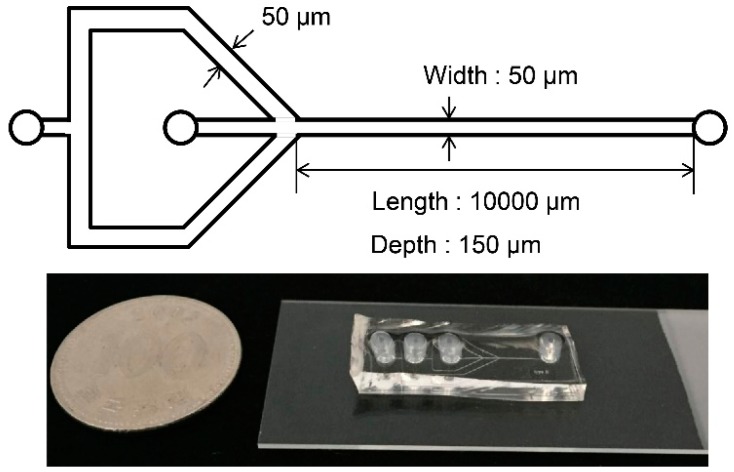
Picture of the fabricated device with detailed dimensions.

The microfluidic device was fabricated by bonding patterned polydimethylsiloxane (PDMS) and a transparent glass slide after treatment with oxygen plasma. A cross-section of the fabrication process of the microfluidic device is presented in Figure 13. First, a 100-µm-thick layer of SU-8 photoresist (SU-8 2050, MicroChem Corp., Westborough, MA, USA) was spin-coated on a flat silicon wafer, and photolithography was conducted to pattern the microfluidic device. The mask used for the photolithography step was designed using AutoCAD software (AutoDesk Inc., San Rafael, CA, USA). After development and hard bake, copper ports were placed onto the SU-8 pattern. Then, the PDMS (Sylgard 184, Dow Corning, Midland, MI, USA) was poured onto the patterned SU-8 mold and cured at 65 °C for 2 h. The PDMS was detached from the SU-8 mold to form the pattern of the microfluidic device on the PDMS. Finally, the patterned PDMS layer was bonded with the glass substrate, after the surface was treated with oxygen plasma (Plasma cleaner PDC-32G, Harrick Plasma Inc., Ithaca, NY, USA) for 2 min. The detailed dimensions and a picture of the fabricated microfluidic device are shown in Figure 14.

### 3.4. Characterization of HPNCs

The HPNCs were characterized using TEM, SEM, and AFM. The TEM samples were prepared by dispersing the HPNCs in THF solvent and placing one drop of the dispersed solution onto carbon-coated copper grids. The grids were then dried at room temperature before the TEM analysis, operated at 300 kV. For the SEM and AFM analysis, the samples were prepared by spin-coating one drop of the dispersed solution on a clean slide glass and then drying them at room temperature before taking the SEM and AFM images.

### 3.5. Sample Preparation for the Measurement of the Refractive Index of HPNCs

Two samples were prepared for measuring the refractive index. One was spin-coated with the photoresist AZ9260 and another was coated with the AZ9260 mixed with the produced HPNCs. The mixture was prepared by mixing the AZ9260 with the 1 wt % HPNCs. The coating was conducted at a rotational speed of 4000 rpm for 1 min. Then, the samples were placed on a hot plate for a soft-bake process at 110 °C for 3 min. The thicknesses of the two samples were measured to be 2.2 ± 0.3 µm and 2.2 ± 0.1 µm, respectively.

## 4. Conclusions

This work presents a convenient and continuous online process to produce HPNCs. They consisted of polymer shells and air cavities to increase the refractive index contrast for the application of OLED. The preparation was obtained by combining the self-assembly of SBR-*b*-PEGMA and the photo-crosslinking process in a flow-focusing microfluidic device. To determine the size, morphology, and hollow structure of the produced the HPNCs, TEM, SEM, and AFM images were taken, and the images showed the successful fabrication of the sphere HPNCs. Also, by controlling the ratio of the inlet flow rates and the concentration of the polymer, it was possible to control the size and morphology of the HPNCs. Furthermore, the photo-crosslinking over the HPNCs of SBR-*b*-PEGMA improved the resistance to organic solvents and water.

Finally, the refractive index of the HPNCs-dispersed layer was decreased compared with that of the layer without HPNCs. This indicates an increase in the index contrast and, as a result, the light trapping of OLED can be reduced. For further research, the highly solvent-resistant HPNCs can be applied to the fabrication of OLED devices with high light extraction efficiency.

## Figures and Tables

**Table 1:** Inlet flow rate and mixing time.

Water (µL/min)	Polymer (µL/min)	Ratio R	*τ*_mix_ (s)
80	40	0.5	0.03
80	60	0.75	0.05
80	80	1	0.07

**Table 2:** Comparison of refractive indices at specific wavelengths.

Wavelength (nm)	Photoresist AZ9260		HPNCs + AZ9260	
	Technical Data	Measured	Calculation	Measured
405	1.6862	1.6864 ± 0.0275	1.6846	1.6269 ± 0.0137
435	1.6722	1.6742 ± 0.0275	1.6707	1.6159 ± 0.0130
